# Novel Molecular Targets in Malignant Diseases of Digestive System 2014

**DOI:** 10.1155/2015/634740

**Published:** 2015-04-29

**Authors:** Chunping Jiang, Youmin Wu, Xiao Xu, Zhongxia Wang

**Affiliations:** ^1^Department of Hepatobiliary Surgery, The Affiliated Drum Tower Hospital of Nanjing University Medical School, Nanjing, Jiangsu 210008, China; ^2^Intra-Abdominal Transplant and Hepatobiliary Surgery, Westchester Medical Center of New York Medical College, Valhalla, NY 10595, USA; ^3^Department of Hepatobiliary and Pancreatic Surgery, First Affiliated Hospital, Zhejiang University School of Medicine, Hangzhou, Zhejiang 310003, China

Despite the continuing advancement in both basic and clinical research, malignant diseases in digestive system remain a serious challenge to human health globally. Study on novel molecular targets and corresponding interventions may help improve the unsatisfactory outcome of digestive cancers. This issue on novel molecular targets in malignant diseases of digestive system consists of seven exciting papers covering gastric cancer (GC), colorectal cancer, hepatocellular carcinoma (HCC), pancreatic cancer, and cholangiocarcinoma.

Chemotherapy is a most frequently used treatment for metastatic or unresectable GC. Unfortunately, the outcome of advanced stage stomach cancer is disappointing. Options of targeted therapy for GC are rather limited. M. Inokuchi and colleagues reviewed the role of fibroblast growth factor receptors (FGFRs) in GC with evidence from currently available literature. The clinical relevance of FGFRs and GC was summarized. Importantly, this review discussed the potentiality of FGFRs as a therapeutic target molecule for GC therapy. This paper will provide us with a comprehensive knowledge on FGFRs and their roles in GC targeted therapy.

Q. Ni et al. presented their study on the association between single nucleotide polymorphisms (SNPs) in microRNAs (miRNAs) and GC susceptibility. This systematic review and meta-analysis synthesized evidence from 12 eligible studies and found potential association between rs2910164 in miR-146a and reduced GC risk. Interestingly, subgroup analyses on rs11614913 in miR-196a3 revealed two-faced effects of this SNP on diffuse and intestinal type GC. The data reported in this study provide us with new insights into the relationship between SNPs in miRNA-coding gene and susceptibility of GC.

N. Piton and colleagues studied the possibility of using immunohistochemistry (IHC) as an alternative to molecular biology methods for detecting KRAS and BRAF mutations in colorectal cancer. This pathology-based study concluded that, using mutation-specific antibody, IHC may also be a reliable diagnostic method for BRAF V600E mutation detection. Due to the lack of specific antibody, KRAS mutation could not be examined efficiently by IHC.

Also by IHC analysis, K. K. Park et al. demonstrated association between human epithelial growth factor receptor 2 (HER2) and mucins/p53 expressions in gastric cancer. Their results indicated MUC2, MUC6, and p53 were significantly correlated with HER2 positivity. HER2 overexpression also independently associated with poor prognosis of gastric cancer. This study will help us understand novel possible mechanism by which HER2 influences the prognosis of GC.

In Q. Ye et al.'s study, the inhibitory effect of Endostar on HCC-induced angiogenesis was reported. As an antiangiogenesis protein drug, Endostar inhibited the migration, proliferation, and tube formation of endothelial cells in response to HCC. Their results indicated that this restructured endostatin protein may help improve the outcome of HCC by targeting angiogenesis, which represents a hallmark of this fatal disease.

Another paper from L. Chen et al. also focused on HCC. Their study demonstrated that the expression of toll-like receptor 3 (TLR3) in HCC cells positively correlated with hepatitis B virus (HBV) infection, interstitial immunoreactive cells infiltration, and cancer cell apoptosis. Activation of TLR3 inhibited the secretion of HBV antigens and induced apoptosis of HCC cells. This manuscript provided initial evidence of TLR3 signaling in HBV immune response in HCC.

S. Bang and colleagues studied the signal transduction pathways involved in antiproliferative effects of a paclitaxel-eluting membrane (PEM) on pancreatic cancer and cholangiocarcinoma. Using nude mice xenograft model, they found that paclitaxel from PEM reduced angiogenesis by inhibiting mammalian target of rapamycin (mTOR) through the regulation of hypoxia inducible factor-1 (HIF-1). PEM also induced tumor cell apoptosis and inhibited tumor-stromal interaction. This study shed light on potential molecular mechanisms underlying this novel drug-eluting membrane and further investigation on this topic may help improve the effect of palliative therapy for inoperable biliary and pancreatic cancers.

In summary, this special issue presents intriguing achievements in the field of novel molecular targets in digestive malignancies.

